# Marked Clinical and Functional Response to Tezepelumab After Failure of Anti-Interleukin-5 (Anti-IL-5) Therapy in Severe Asthma

**DOI:** 10.7759/cureus.103648

**Published:** 2026-02-15

**Authors:** Makoto Fujimoto, Toyoshi Yanagihara, Yuka Sakaki, Masaki Fujita

**Affiliations:** 1 Department of Respiratory Medicine, Fukuoka University Hospital, Fukuoka, JPN

**Keywords:** airflow limitation, biologics, il-5 antibody, severe asthma, tezepelumab

## Abstract

Biologics targeting type 2 inflammation, including anti-interleukin-5 (anti-IL-5) antibodies, have improved outcomes in severe asthma, but some patients remain symptomatic with apparently persistent severe airflow limitation. We report an 86-year-old woman with long-standing severe asthma who initially had high blood eosinophil counts and serum immunoglobulin E (IgE) levels. She responded to mepolizumab for years, while airflow limitation persisted. She developed an exacerbation seven years after mepolizumab initiation despite ongoing mepolizumab and inhaled triple therapy, while type 2 biomarkers normalized. She continued to experience dyspnea with triggers such as cold air and pollen. After switching from mepolizumab to tezepelumab, her symptoms rapidly resolved; forced expiratory volume in one second (FEV₁) increased from 0.64 L (45.7% predicted) to 1.45 L (103% predicted) within two months, and the concave flow-volume loop became almost normal. This case suggests that chronic airflow limitation in severe asthma is not always irreversible and that switching to tezepelumab may induce dramatic functional recovery even after anti-IL-5 therapy.

## Introduction

Severe asthma is a heterogeneous condition affecting approximately 5-10% of patients with asthma and remains uncontrolled despite high-dose inhaled corticosteroids and additional therapies. The introduction of biologics targeting type 2 inflammation, such as anti-immunoglobulin E (anti-IgE), anti-interleukin-5 (anti-IL-5), anti-IL-5 receptor alpha (anti-IL-5Rα), and anti-interleukin-4 receptor alpha (anti-IL-4Rα) antibodies, has substantially improved outcomes in many patients [1]. However, a proportion of patients continue to experience frequent exacerbations and poor symptom control even with these agents.

Tezepelumab is a novel monoclonal antibody targeting thymic stromal lymphopoietin (TSLP), an upstream epithelial cytokine that drives both type 2 and non-type 2 inflammatory pathways [2]. Clinical trials and real-world studies have demonstrated its efficacy in reducing exacerbations and improving asthma control, regardless of biomarker status. While the efficacy of tezepelumab has been shown in biologic-naïve patients, real-world data on patients switching to tezepelumab after inadequate response to other biologics remain limited. Particularly, few case reports have documented marked reversibility of persistent severe airflow obstruction following such a switch.

Here, we report a case of a patient with severe bronchial asthma and chronic, apparently irreversible airflow limitation who achieved dramatic improvement in both symptoms and lung function after switching from mepolizumab to tezepelumab. This case highlights the potential role of tezepelumab in treatment-resistant asthma where conventional biologics have failed.

## Case presentation

**Figure 1:**
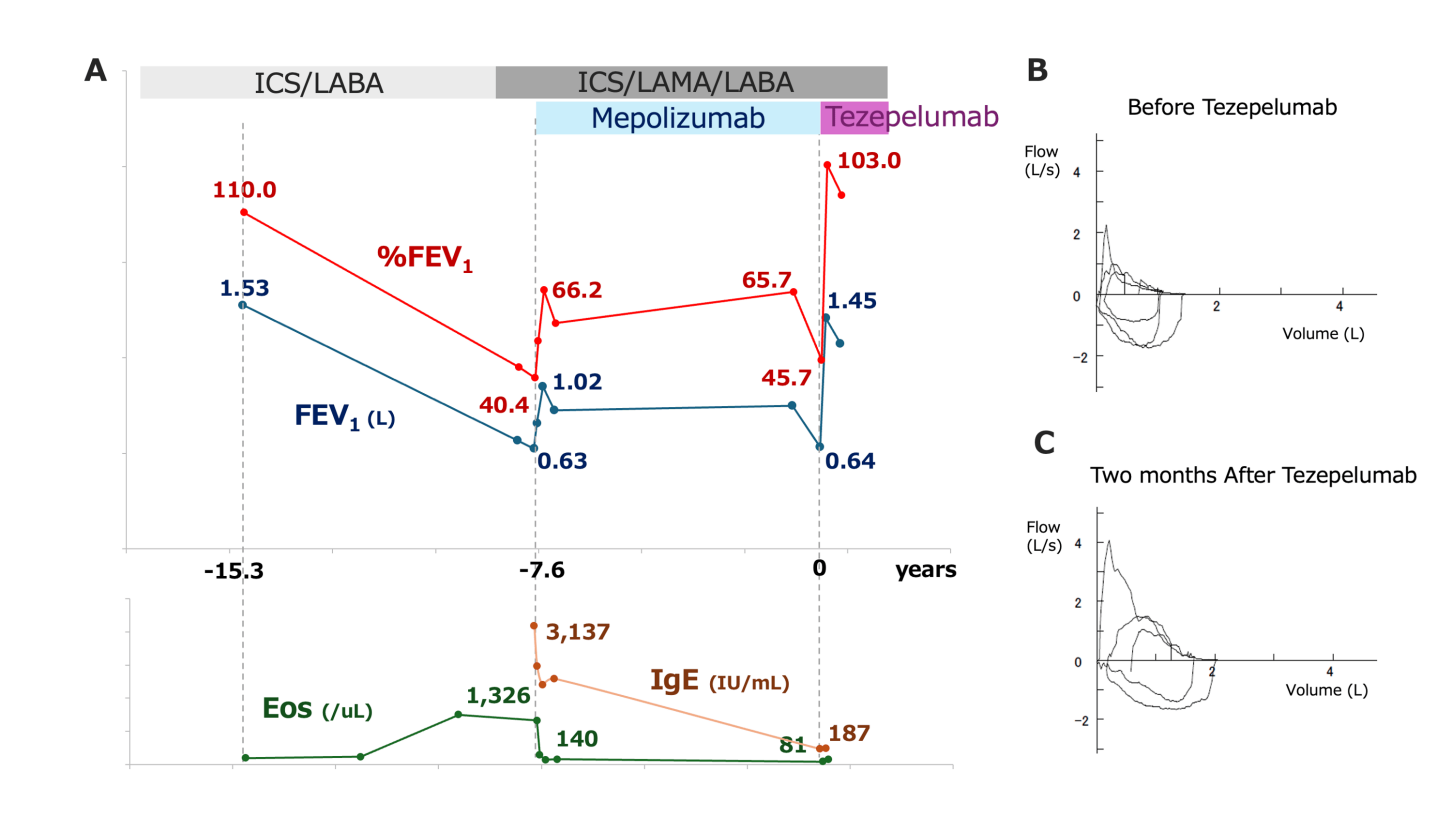
Longitudinal changes in lung function, biomarkers, and flow-volume curves before and after switching from mepolizumab to tezepelumab. (A) Time course of FEV₁ (L) (blue) and %FEV₁ (red) during treatment with ICS/LABA and ICS/LAMA/LABA plus mepolizumab and after switching to tezepelumab, together with peripheral blood eosinophil counts (Eos, green) and serum IgE levels (brown). Despite prior mepolizumab therapy, FEV₁ and %FEV₁ progressively declined, with recovery only after the initiation of tezepelumab. (B) Flow-volume curve obtained immediately before starting tezepelumab, showing marked, almost fixed airflow obstruction. (C) Flow-volume curve two months after starting tezepelumab, demonstrating the near-complete normalization of expiratory flow and resolution of airflow limitation. ICS: inhaled corticosteroid; LABA: long-acting β₂-agonist; LAMA: long-acting muscarinic antagonist; FEV₁: forced expiratory volume in one second; Eos: eosinophils; IgE: immunoglobulin E

An 86-year-old woman, a lifelong never-smoker, presented with dyspnea and cough. She had been diagnosed with bronchial asthma in her 40s and had been followed at our hospital for refractory disease for more than nine years. She reported symptom worsening with cold air and pollen exposure. A detailed history did not identify any other specific triggers or recent environmental changes associated with symptom deterioration. Comorbidities included hypertension, osteoporosis, reflux esophagitis, and chronic rhinosinusitis. Because her asthma remained poorly controlled on inhaled corticosteroid/long-acting β₂-agonist (ICS/LABA), treatment had been escalated to inhaled triple therapy (ICS/LABA/long-acting muscarinic antagonist (LAMA)), and mepolizumab was initiated 7.6 years before presentation (Figure 1A). At that time, she had marked eosinophilia (1,326/μL) and elevated total IgE (3,137 IU/mL) with severe airflow limitation (% predicted forced expiratory volume in one second (%FEV₁) 40.4%). Her exacerbations were well controlled for several years after the introduction of mepolizumab, whereas airflow limitation persisted, with %FEV₁ remaining around 60%.

**Figure 2:**
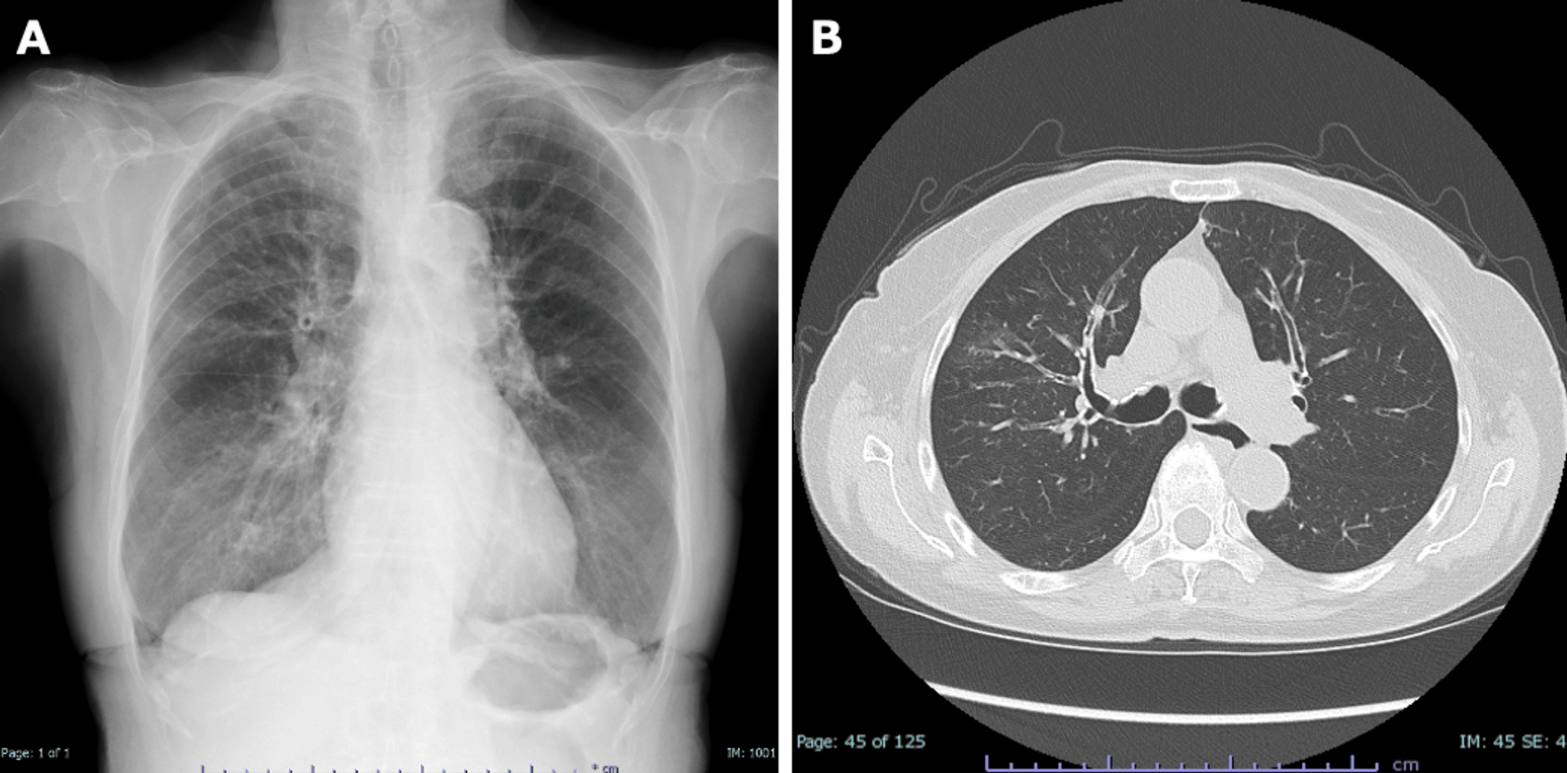
Chest radiograph and high-resolution computed tomography findings. (A) Posteroanterior chest radiograph showing subtle ground-glass opacity in the right upper lung field. (B) Axial high-resolution computed tomography image demonstrating bilateral bronchial wall thickening and scattered faint ground-glass opacities around the right upper lobe bronchi.

However, in the winter of the index year, she developed recurrent asthma symptoms requiring oral prednisolone, followed by persistent wheeze and exertional dyspnea. On presentation in spring, she was ambulatory with an oxygen saturation of 92% on room air and diffuse bilateral wheezes. The Asthma Control Test (ACT) score was 5. Laboratory tests showed a low eosinophil count (81/µL), total IgE of 187 IU/mL, and C-reactive protein (CRP) of 0.04 mg/dL (Table 1). Allergen-specific IgE testing was positive for Japanese cedar pollen (class 3) and weakly positive for cypress and *Aspergillus*. Chest radiography showed subtle ground-glass opacity in the right upper lung field. High-resolution computed tomography (HRCT) demonstrated bilateral bronchial wall thickening with scattered faint ground-glass opacities around the right upper lobe bronchi, without definite bronchiectasis on standard radiologic criteria (Figure 2A, 2B). Spirometry showed severe persistent airflow limitation with a markedly concave expiratory flow-volume loop. FEV₁ was 0.64 L (45.7% predicted) (Figure 1B and Table 2). She required several short courses of oral corticosteroids for symptom worsening; however, she did not require hospitalization or inpatient treatment during this period.

**Table 1:** Laboratory investigations at tezepelumab initiation. Laboratory results obtained at the time of tezepelumab initiation. WBC: white blood cell count; RBC: red blood cell count; Hb: hemoglobin; Plt: platelet count; TP: total protein; Alb: albumin; CRP: C-reactive protein; AST: aspartate aminotransferase; ALT: alanine aminotransferase; BUN: blood urea nitrogen; Cr: creatinine; Na: sodium; K: potassium; IgE: immunoglobulin E

Test	Value	Reference range
WBC (/μL)	3,700	3,300-8,600
RBC (10^4^/μL)	451	435-555
Hb (g/dL)	13.6	13.7-16.8
Plt (10^3^/μL)	146	158-348
TP (g/dL)	7.9	6.6-8.1
Alb (g/dL)	3.8	3.8-5.3
CRP (mg/dL)	0.04	0.4-1.5
AST (U/L)	24	13-30
ALT (U/L)	18	10-42
BUN (mg/dL)	14	8-20
Cr (mg/dL)	0.52	0.65-1.07
Na (mEq/L)	142	138-145
K (mEq/L)	4.6	3.6-4.8
IgE (IU/mL)	187	<232

**Table 2:** Pulmonary function test before and after tezepelumab initiation. Pulmonary function parameters are shown at baseline (before tezepelumab) and after tezepelumab treatment. Values are presented as absolute measurements (L or L/s) and percent predicted (%pred), as indicated. SVC: slow vital capacity; ERV: expiratory reserve volume; IRV: inspiratory reserve volume; TV: tidal volume; IC: inspiratory capacity; FVC: forced vital capacity; FEV1.0: forced expiratory volume in one second; FEV1.0%: FEV1/FVC ratio; PEFR: peak expiratory flow rate; V50 and V25: maximal expiratory flow at 50% and 25% of FVC, respectively; MMF: maximal mid-expiratory flow (forced expiratory flow 25-75%)

	Before tezepelumab	After tezepelumab
SVC	1.50 L	2.32 L
%SVC	72.50%	112.10%
ERV	0.33 L	0.66 L
IRV	0.26 L	0.73 L
TV	0.91 L	0.93 L
IC	1.17 L	1.66 L
FVC	1.55 L	2.22 L
%FVC	81.20%	116.20%
FVC Z-score	-1.61	-0.48
FEV1.0	0.64 L	1.45 L
%FEV1.0	45.70%	103.60%
FEV1.0 Z-score	-3.88	0.41
FEV1.0%	41.29%	65.32%
PEFR	2.26 L/s	4.06 L/s
%PEFR	52.70%	94.60%
V50	0.23 L/s	1.06 L/s
%V50	11.60%	53.50%
V25	0.08 L/s	0.14 L/s
%V25	17.80%	31.10%
MMF	0.18 L/s	0.54 L/s
%MMF	11.20%	33.80%

Given uncontrolled symptoms and progressive, apparently persistent severe airflow obstruction despite inhaled triple therapy (Trelegy Ellipta 200) plus mepolizumab, we switched from mepolizumab to tezepelumab. Given low blood eosinophils and normalized total IgE at relapse, dupilumab was considered; however, we favored tezepelumab because its upstream TSLP blockade is effective across biomarker strata and can modulate both type 2 and non-type 2 pathways, which was clinically relevant in this context. Symptoms improved promptly, with the ACT score increasing to 25. FEV₁ increased to 1.45 L (103% predicted) with the near-normalization of the flow-volume curve within two months (Figure 1C). The patient has remained in clinical remission thereafter.

## Discussion

This case suggests that chronic airflow limitation in severe asthma is not always irreversible and may improve when upstream pathways of airway inflammation are adequately targeted. In a recent retrospective study of patients with severe asthma who were switched from other biologics to tezepelumab, 77% were free from exacerbations at 12 weeks, and 64% achieved a clinically important improvement in ACT score [3]. However, the median change in FEV₁ was minimal (+40 mL), and overall lung function benefit appeared limited. Notably, within this cohort, two of 22 patients showed an increase in FEV₁ of ≥200 mL, indicating that a subset of "super-responders" exists even among biologic-experienced patients. Our case is consistent with this concept and illustrates that, in selected patients, switching to tezepelumab can lead not only to symptom control but also to the near-normalization of previously persistent severe airflow limitation.

At baseline before mepolizumab administration, our patient had marked eosinophilia and elevated IgE, indicating a predominant type 2 inflammatory phenotype, and responded initially to mepolizumab. When symptoms later recurred and became treatment-refractory, blood eosinophils and IgE were no longer elevated. This change raises the possibility that non-type 2 pathways, including neutrophilic inflammation, had become more prominent. Although dupilumab was a potential alternative, we favored tezepelumab because relapse occurred with low eosinophils and normalized IgE, raising concern that a strictly type 2-high pattern was no longer predominant, and because upstream TSLP inhibition offers a biomarker-agnostic strategy that may better address mixed inflammatory endotypes. In such a setting, further intensification of anti-IL-5 or anti-IL-4/IL-13 therapy may have limited benefit, whereas an upstream intervention that modulates multiple downstream pathways may be more appropriate.

Tezepelumab blocks TSLP, an epithelial cytokine released in response to triggers such as viral infections, allergens, cold air, and other environmental stimuli [4,5]. Our patient reported cold air and pollen as clear triggers of exacerbation, which supports a major contribution of epithelial activation to her disease. By interrupting the TSLP pathway, tezepelumab can attenuate both type 2 and non-type 2 inflammatory cascades and may also reduce airway hyperresponsiveness. In our case, this translated into the abolition of chronic airflow obstruction despite many years of disease, suggesting that at least part of the "persistent severe airflow limitation" component was still modifiable.

Several potential confounders should be considered when interpreting the marked spirometric improvement, including concurrent changes in inhaled or oral corticosteroid exposure, other controller medications, adherence, or inhaler technique, as well as effort-dependent variability and seasonal fluctuation. In this case, inhaled triple therapy (Trelegy Ellipta 200) was continued without dose or device changes, and adherence and inhaler technique remained unchanged; moreover, the patient had performed spirometry repeatedly for >15 years, making an abrupt learning effect unlikely. While causality cannot be proven in a single-patient observation, the improvement was temporally linked to tezepelumab initiation and remained stable thereafter, as shown in the revised clinical course figure.

As a limitation, *Aspergillus*-specific IgG was not assessed in this case. Accordingly, allergic bronchopulmonary aspergillosis (ABPA) could not be definitively excluded by a complete serologic assessment, although the overall clinical and radiologic findings were not strongly suggestive. In addition, pre- and post-bronchodilator spirometry was not performed, so bronchodilator reversibility could not be evaluated, which limits the physiologic interpretation of the marked improvement.

Taken together with previous real-world data on super-responders, our observations support considering tezepelumab as a therapeutic option in patients with severe asthma who remain symptomatic with persistent airflow limitation despite IL-5-targeted biologic therapy.

## Conclusions

Switching from mepolizumab to tezepelumab was followed by rapid symptom resolution and the near-normalization of spirometry in an elderly patient with long-standing severe asthma. This exceptional response suggests that persistent severe airflow limitation may be more modifiable than expected in selected patients and supports considering tezepelumab when anti-IL-5 therapy provides inadequate control. Further real-world studies are needed to identify predictors of such "super-responder" profiles and to clarify the mechanisms of functional recovery.
